# Hepatic lymphocytes involved in the pathogenesis of pediatric and adult non-alcoholic fatty liver disease

**DOI:** 10.1038/s41598-021-84674-z

**Published:** 2021-03-04

**Authors:** Victoria Cairoli, Elena De Matteo, Daniela Rios, Carol Lezama, Marcela Galoppo, Paola Casciato, Eduardo Mullen, Cecilia Giadans, Gustavo Bertot, María Victoria Preciado, Pamela Valva

**Affiliations:** 1 Multidisciplinary Institute for Investigation in Pediatric Pathologies (IMIPP), CONICET-GCBA, Laboratory of Molecular Biology, Pathology Division, Ricardo Gutiérrez Children’s Hospital, Gallo 1330, C1425EFD Buenos Aires, CABA Argentina; 2 Liver Unit, Ricardo Gutiérrez Children’s Hospital, C1425EFD Buenos Aires, CABA Argentina; 3 Liver Unit, Italian’s Hospital of Buenos Aires, C1199ABH Buenos Aires, CABA Argentina; 4 Pathology Division, Italian’s Hospital of Buenos Aires, C1199ABH Buenos Aires, CABA Argentina; 5 H.A. Barceló Foundation-Medicine University, C1425EFD Buenos Aires, CABA Argentina

**Keywords:** Non-alcoholic fatty liver disease, Non-alcoholic steatohepatitis, Immunopathogenesis, Adaptive immunity

## Abstract

The immune response is critical in NAFLD pathogenesis, but the liver infiltrate’s composition and the role of each T cell population is still up for debate. To characterize liver pathogenesis in pediatric and adult cases, frequency and localization of immune cell populations [Cytotoxic T Lymphocytes (CD8+), T helper Lymphocytes (CD4+), Regulatory T lymphocytes (Foxp3+) and Th17 (IL-17A+)] were evaluated. In portal/periportal (P/P) tracts, both age groups displayed a similar proportion of CD8+ and CD4+ lymphocytes. However, comparable Foxp3+ and IL-17A+ cell frequencies were observed in pediatric cases, meanwhile, in adults Foxp3+ was higher than IL-17A+ cells. Interestingly, IL-17A+ lymphocytes seemed to be nearly exclusive of P/P area in both age groups. In intralobular areas, both pediatric and adult cases showed CD8+ lymphocytes predominance with lower frequencies of CD4+ lymphocytes followed by Foxp3+ . Severe inflammation was associated with higher intralobular Foxp3+ lymphocytes (*p* = 0.026) in children, and lower P/P Foxp3+ and higher IL-17A+ lymphocytes in adults. All cases with fibrosis ≥ 2 displayed P/P low Foxp3+ and high IL-17A+ lymphocyte counts. Pediatric cases with worse steatosis showed high P/P CD4+ (*p* = 0.023) and intralobular CD8+ (*p* = 0.027) and CD4+ cells (*p* = 0.012). In NAFLD cases, the lymphocyte liver infiltrate composition differs between histological areas. Treg and Th17 balance seems to condition damage progression, denoting their important role in pathogenesis.

## Introduction

The obesity epidemic that promotes premature development of metabolic syndrome is a global concern since it significantly increases the risk for liver disease early in life. Non-alcoholic fatty liver disease (NAFLD) represents a serious nutritional worry which arises from the high prevalence of overweight and obesity because it is the most common form of chronic liver illness in children and adults^1^. It is estimated that one-quarter of the world's adult population is affected, being the global prevalence as high as one billion^2^. Epidemiological data on pediatric NAFLD are still scarce, but the overall prevalence of the disease is estimated to be 7.6% (95% CI 5.5–10.3%) in the general pediatric population and 34.2% (95% CI 27.8–41.2%) in children who are obese^3–5^. NAFLD is characterized by an excessive hepatic fat accumulation and includes two conditions with different prognoses: non-alcoholic fatty liver (NAFL) and non-alcoholic steatohepatitis (NASH)^6^. Notably, the patient management and therapy planning rely on the distinction between these two disease forms, since NAFL does not adversely affect the long-term outcome, whereas NASH has a more rapid progression to advanced liver disease^6^. The field of liver transplantation will be probably overloaded in the near future due to the emergence NASH, NASH-related cirrhosis, decompensated liver disease and hepatocellular carcinoma (HCC)^7^. Today NASH is the third most common indication for liver transplantation, but it is steadily climbing^8^.

The spectrum of histological lesions in individuals with NAFLD ranges from simple steatosis to NASH. There are three situations that define NAFL, namely a) steatosis, b) steatosis with lobular or portal inflammation in the absence of ballooning, or c) steatosis with ballooning but without inflammation. On the other hand, the diagnosis of NASH requires an accurate histopathological evaluation to assess the presence of steatosis, ballooning and lobular inflammation^9^. Besides, although the presence of perisinusoidal fibrosis is also a frequent histological sign, it does not represent a criterion that has diagnostic weight. The fibrosis progression correlates with liver-related outcomes and death; therefore it is the most significant prognostic factor^10^.

It is still a matter of debate which are the complex pathophysiological pathways that participate in the progression from simple steatosis to NASH with the consequent liver damage, but it is taken for sure the existence of a crosstalk between various metabolically active tissues^11^. In this metabolic disease, insulin resistance, hepatic fatty acid accumulation, oxidative stress, mitochondrial dysfunction, and a systemic proinflammatory state are involved in liver damage and inflammation, in consequence, NASH could be assumed as a multifactorial condition. Concerning these multiple hits, they may take place in parallel rather than consecutively, therefore the term "multiple parallel hits hypothesis" was coined to describe NASH pathogenesis. Currently, NAFLD is considered as a disease in which multiple other organ systems contribute to the pathogenesis of liver inflammation^12^. The immune response plays a decisive role in the pathogenesis of NASH since hepatic inflammation is one of the most pronounced features^13–15^. T cells [CD8+ cytotoxic T lymphocytes (CTL) and various CD4+ T helper subsets] seem to participate in NAFLD pathogenesis^11^. It is known that liver inflammation is triggered by lipid deposits enlargement in hepatocytes which leads to the production of pro-inflammatory cytokines^16^. Just as described in alcohol-associated liver disease, chronic viral hepatitis, autoimmune liver diseases, and hepatocellular carcinoma^13,17–19^; regulatory T (Treg) cells are crucial participants that regulate inflammation in NASH, while CD4+ T helper 17 (Th17) cells might functionally oppose Treg-function^16^. Therefore, the balance between tolerance and elicitation of immune response is conditioned by the reciprocal relationship between Treg and Th17 cells^17^. Besides, it has been also suggested that Th17 cells and CTLs worsen liver damage and fibrosis progression in NASH^11,16^. Liver immune microenvironment composition and cell distribution may be affected by certain pathological processes^20,21^; however, due to the limitations and difficulty to obtain human liver samples and the use of animal models the accurate role of the T cell is yet controversial. As mentioned above, while sustained intrahepatic inflammation is considered critical in the progression from simple steatosis to NASH; it is still not clear the infiltrate composition and the role of each cell population in the pathogenesis.

Finally, the study of patient’s samples instead of animal models is essential to explore different aspects of NAFLD in its actual context even taking into account interpatient variability. The inclusion of pediatric cases in the present study further enriches the analysis. It arises from literature that most NAFLD studies relied solely on blood samples; thus, the description of the liver microenvironment provides a more comprehensive picture of lymphocyte population in relation with liver injury and it contributes a valuable insight into NAFLD pathogenesis. Consequently, in the light of these considerations, the aim of the present study was to evaluate the presence and localization of certain cell populations of the liver microenvironment together with their interrelationship to understand their participation in the NAFLD pathogenesis.

## Results

### Clinical and liver biopsy findings

Sixty NAFLD cases were enrolled, of which 26 were children (Table 1).

**Table 1:** Clinical and histological patients feature.

Factor	Patients	
	Pediatric	Adult
**Age (years)** median (min.–max.)	11.5 (4–17)	50 (28–72)
**Gender** [male, %(n/total)]	65.38 (17/26)	52.94 (18/34)
**Clinical and serological characteristics**		
BMI		
Overweighed %(n/total)	11.53 (3/26)	29.41 (24/34)
Obese %(n/total)	76.96(20/26)	70.59 (10/34)
Transaminases		
ALT (IU/l) median (min.–max.)	31.5 (16–216)	80 (31–279)
Elevated %(n/total)	50 (13/26)	94.12 (32/34)
AST (IU/l) median (min.–max.)	40.5 (15–213)	48 (22–208)
Elevated %(n/total)	42.31 (11/26)	61.76 (21/34)
AST/ALT ratio median (min.–max.)	0.75(0.385–1.409)	0.63 (0.32–1.07)
Lipid profile median (min.–max.)		
Cholesterol mg/dl	154.5 (88–224)	207 (126–327)
Triglycerides mg/dl	100 (28–212)	166 (60–465)
Histological characteristics*		
Steatosis%(n/total)		
0	–	–
1	15.38 (4/26)	17.65 (6/34)
2	19.23 (5/26)	26.47 (9/34)
3	65.39 (17/26)	55.88 (19/34)
Lobular inflammation%(n/total)		
0	3.85 (1/26)	17.65 (6/34)
1	80.77 (21/26)	64.70 (22/34)
2	7.69 (2/26)	17.65 (6/34)
3	7.69 (2/26)	–
Ballooning %(n/total)		
0	–	11.76 (4/34)
1	69.23 (18/26)	64.71 (22/34)
2	30.77 (8/26)	23.53 (8/34)
NAFLD activity score %(n/total)		
≤ 2	–	8.82 (3/34)
3–4	23.08 (6/26)	38.24 (13/34)
≥ 5	76.92 (20/26)	52.94 (18/34)
Fibrosis %(n/total)		
0	–	67.65 (23/34)
1	15.38 (4/26)	14.71 (5/34)
2	23.08 (6/26)	11.76 (4/34)
3	57.69 (15/26)	5.88 (2/34)
4	3.85 (1/26)	–
n	26	34

*BMI*: Body Mass Index (kg/m^2^), in pediatric patients the values defined by the Argentine Society of Pediatrics were used to determine overweight and obesity (https://www.sap.org.ar/uploads/consensos/obesidad-gu-iacuteas-para-su-abordaje-cl-iacutenico-2015.pdf). In the case of adult patients: normal weight (< 25.0 kg/m^2^), overweight (25.0–29.9 kg/m^2^) and obesity (≥ 30 kg/m^2^). *ALT* alanine aminotransferase; *AST* aspartate aminotransferase. Normal ALT and AST levels for pediatric patients were ≤ 32 and ≤ 48 IU/L, respectively when testing was done at 37 °C. Normal ALT and AST levels for adult patients were ≤ 40 and ≤ 42 IU/L, respectively when testing was done at 37 °C. The normal ranges for cholesterol and triglyceride were 120–219 mg/dl and 31–119 mg/dl, respectively. *Steatosis Grade: score 0 (< 5%cells), 1 (5–33%), 2 (33–66%) and 3 (> 66%); lobular inflammation: score 0 (0 foci), 1 (< 2 foci), 2 (2–4 foci) and 3 (> 4 foci); ballooning grade: score 0 (none), 1 (few ballooning cells) and 2 (many cells/prominent cells); fibrosis stage: score 1 (a, b = mild (1a)/ moderate (1b) zone 3 perisinusoidal fibrosis; 1c = only portal fibrosis); 2 (zone 3 and portal/ periportal fibrosis), 3 (bridging fibrosis) and 4 (cirrhosis).

Concerning histological parameters, both age groups showed a predominance of grade 3 steatosis, lobular inflammation grade 1 and ballooning grade1. Interestingly, fibrosis showed a different pattern between the two age groups. The prevalence of significant fibrosis (F≥ 2) in the pediatric cohort was 84.64% (22/26) while in adults it was 17.65% (6/34).

In accordance with the report of the NASH Clinical Research Network classification, none of the pediatric patients were classified as “not NASH or simple steatosis”, 76.92% (20/26) were diagnosed as “definitive NASH” and 23.08% (6/26) as “borderline NASH”. Meanwhile, 52.94% (18/34) of adult patients were diagnosed as ‘definitive NASH’, 38.24% (13/34) as ‘borderline NASH’, and 8.82% (3/34) as ‘not NASH’.

## Characterization of lymphocyte populations in the liver microenvironment

**Figure 1:**
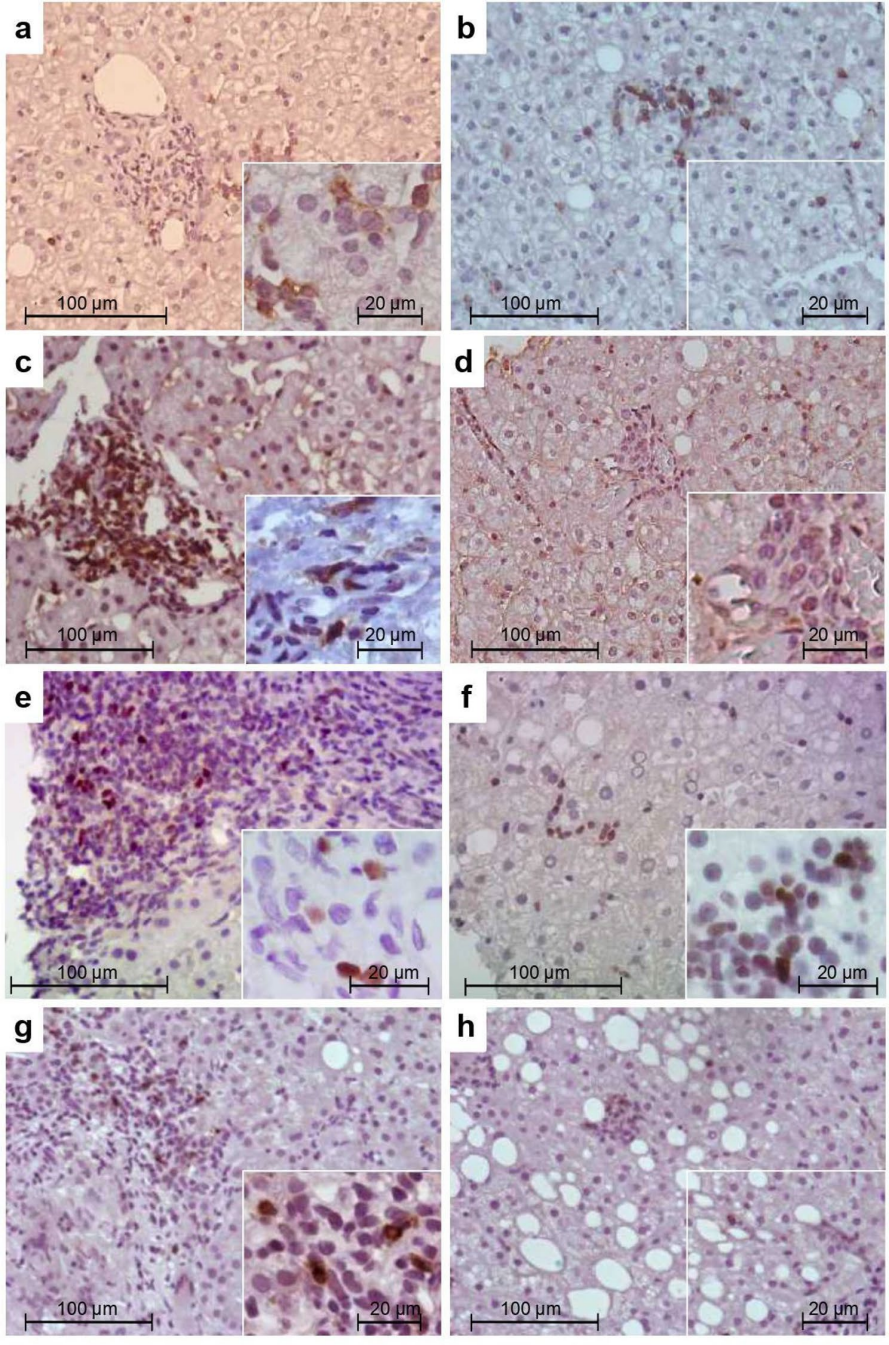
Immunostaining of liver infiltrating lymphocyte populations on formalin-fixed liver biopsies. CD8+ (**a**,**b**), CD4+ (**c**,**d**), Foxp3+ (**e**,**f**) and IL-17A+ (**g**,**h**) cells. Representative images of portal-periportal (**a**,**c**,**e**,**g**) and intralobular (**b**,**d**,**f**,**h**) area.

The immune cell liver microenvironment was assessed by an immunohistochemical approach on liver biopsies from pediatric and adult NAFLD patients by means of CD8+ and CD4+ cells as well as CD4+ cell-subsets detection. All the studied populations were predominantly identified in the portal-periportal infiltrate areas while only scattered lymphocytes were observed in the intralobular region (Fig. 1a–h).

Concerning the infiltrating cell frequencies in the portal-periportal areas (Supplementary Fig. 1a, b, d, e), it displayed a profile with a similar proportion of CD8+ and CD4+ cells both in pediatric and adult NAFLD cases (Supplementary Fig. 1a and d). When analyzing the CD4+ subsets, similar frequencies of Foxp3+ and IL-17A+ cells were observed in pediatric cases (Supplementary Fig. 1a). Meanwhile, in adult samples a profile with frequencies of Foxp3+ cells higher than IL-17A+ cells was observed (Supplementary Fig. 1d). It’s remarkable that in a more detailed analysis on a case-by-case basis, a clear Foxp3+ predominance over IL-17A+ lymphocytes in adult NAFLD cases was observed; however, this pattern was not clearly represented in pediatric cases (Supplementary Fig. 2 PANEL 1). In the intralobular area, both pediatric and adult cases showed CD8+ lymphocyte predominance with lower frequencies of CD4+ lymphocytes followed by Foxp3+ cells (Supplementary Fig. 1c and f). An interesting finding concerning IL-17A+ lymphocyte population was its asymmetric tissue distribution with exclusive labeling in the portal-periportal area in most cases (Supplementary Fig. 1a, c, d, f). Table 2 shows each cell population frequency in both age groups, pondered in both portal-periportal and intralobular areas.

**Table 2:** Lymphocyte frequencies in NAFLD liver samples.

	Pediatric patients	Adult patients
**Portal and periportal area**		
Total lymphocytes	105 (25–314)	88 (15–538)
CD8^+^ cells	0.482 (0.146–0.704)	0.423 (0.016–0.689)
CD4^+^cells	0.353 (0–0.806)	0.441(0–0.840)
Foxp3^+^ cells	0.035 (0–0.112)	0.069 (0–0.605)
IL-17A^+^ cells	0.030 (0–0.123)	0.042 (0.016–0.225)
IL-17A^+^/Foxp3^+^ cell ratio	1.242 (0–3.446)	0.329 (0–1.600)
**Intralobular area**		
CD8^+^ cells	8 (0–61)	31 (0–108)
CD4^+^cells	1 (0–22)	16 (0–67)
Foxp3^+^ cells	0 (0–7)	3.5 (0–23)
IL-17A^+^ cells	0 (0–6)	0 (0–4)
IL-17A^+^/Foxp3^+^ cell ratio	0 (0–1.2)	0 (0–0.500)

Results are expressed as median (min.-max.)

Considering NAFLD histological classification, the frequency of each lymphocyte population was compared among subgroups; namely, ‘not NASH’, ‘borderline NASH’, ‘definitive NASH’. Interestingly, in both age groups the frequency profile for each lymphocyte population and subpopulation remained the same no matter which histological category was considered (Supplementary Fig. 1a, c, d,f). The same was observed when considering total lymphocytes, although, due to the few ‘not NASH’ adult cases we could not confirm the tendency to lower counts (Supplementary Fig. 1e). Despite that, the frequency of the cell populations studied in the portal-periportal area did not show differences among the histological subgroups; the total lymphocyte count was lower in the “not NASH” adult subgroup (Supplementary Fig. 1e).

Since it has been demonstrated that the imbalance between Treg and Th17 cells might play an important role in the course of inflammatory diseases, we evaluated IL-17A+/Foxp3+ cell ratio in the studied cases. The IL-17A+/Foxp3+ cell ratio profile showed a sustained increment from "not NASH" to "NASH" which is clearly evident in adults (Supplementary Fig. 2 PANEL 2).

## Intrahepatic inflammatory infiltrate related to liver damage

**Figure 2:**
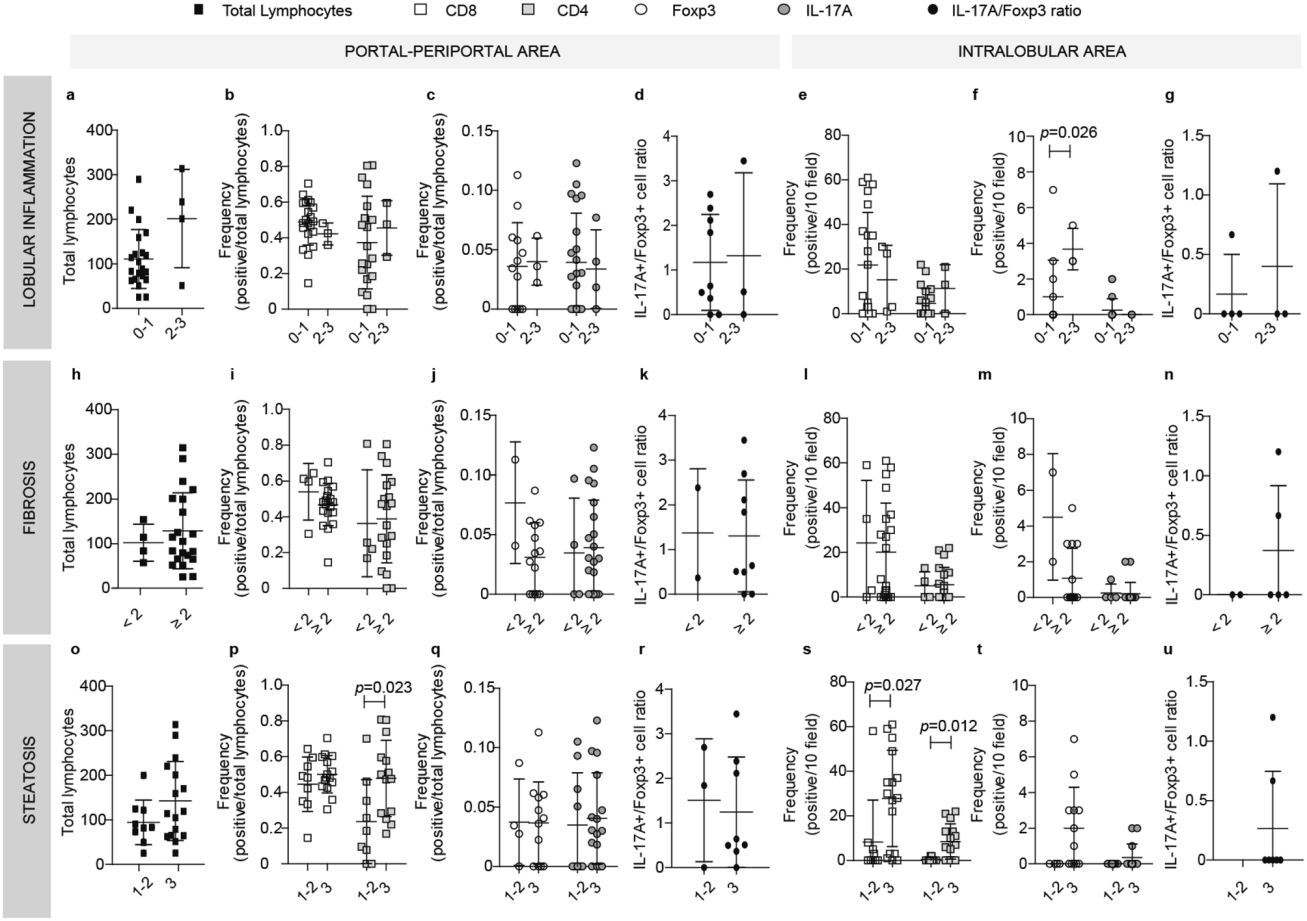
Relationship between intrahepatic infiltrate and liver damage in NAFLD pediatric cases. Lymphocyte quantification related to lobular inflammation (**a**–**g**), fibrosis (**h**–**n**) and steatosis (**o**–**u**) severity. Total lymphocytes (**a**,**h**,**o**), CD8+ and CD4+ (**b**,**i**,**p**), Foxp3+ and IL-17A+ cell frequency (**c**,**j**,**q**) and IL-17A+ /Foxp3+ cell ratio (**d**,**k**,**r**) in portal-periportal area. CD8+ and CD4+ (**e**,**l**,**s**), Foxp3+ and IL-17A+ cell frequency (**f**,**m**,**t**) and IL-17A+ /Foxp3+ cells ratio (**g**,**n**,**u**) in intralobular area.

**Figure 3:**
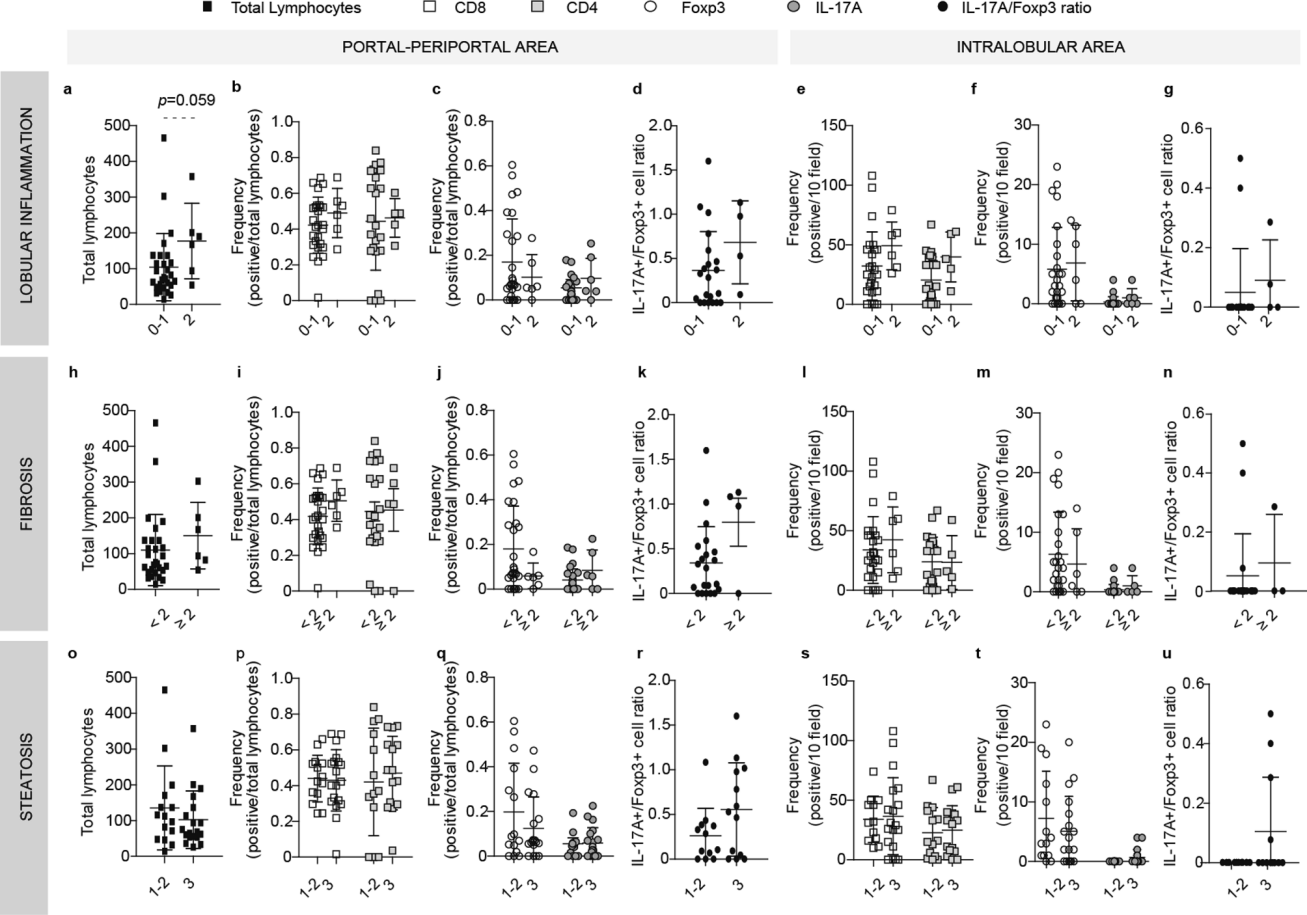
Relationship between intrahepatic infiltrate and liver damage in NAFLD adult cases. Lymphocyte quantification related to lobular inflammation (**a**–**g**), fibrosis (**h**–**n**) and steatosis (**o**–**u**) severity. Total lymphocytes (**a**,**h**,**o**), CD8+ and CD4+ (**b**,**i**,**p**), Foxp3+ and IL-17A+ cell frequency (**c**,**j**,**q**) and IL-17A+ /Foxp3+ cell ratio (**d**,**k**,**r**) in portal-periportal area. CD8+ and CD4+ (**e**,**l**,**s**), Foxp3+ and IL-17A+ cell frequency (**f**,**m**,**t**) and IL-17A+ /Foxp3+ cells ratio (**g**,**n**,**u**) in intralobular area.

The role of the intrahepatic infiltrate in chronic liver injury was analyzed according to three histological features, namely, inflammation (Figs. 2a–g and 3a–g), fibrosis (Figs. 2h–n and 3h–n), and steatosis (Figs. 2o–u and 3o–u).

Concerning lobular inflammation, despite the low number of severe cases in both age groups, the total portal-periportal lymphocyte count showed a profile tending to higher values in those cases with worse histological damage (Figs. 2a and 3a). In pediatric cases the frequency of CD8+, CD4+, Foxp3+ and IL-17A+ cells in the portal-periportal area did not show differences according to the inflammation degree (Fig. 2b,c); while, in the intralobular area those cases with higher inflammation seem to have lower CD8+ lymphocyte values and significantly higher Foxp3+ cell frequencies (*p*=0.026) (Fig. 2e,f). Instead, in adult cases, the portal-periportal Foxp3+ and IL-17A+ cells displayed inverted profiles. Cases with more severe inflammation presented low frequencies of Foxp3+ and high of IL-17A+ cells. Finally, in this age group no lymphocyte population depicted an association with the inflammation severity in the intralobular area (Fig. 3e–g).

As regards fibrosis stages, in pediatric cases portal-periportal total lymphocyte count displayed higher values according to fibrosis severity, but the difference was statistically significant only in advanced fibrosis (Fig. 2h and Supplementary Fig. 3). Foxp3+ cells and IL-17A+ cells depicted altered frequencies in both portal-periportal and intralobular areas, namely, lower Foxp3+ cells and higher IL-17A+ cell counts in those cases with fibrosis ≥ 2 (Fig. 2j,m). Moreover, this inverted profile between Foxp3+ and IL-17A+ cells described in children was also observed in the portal-periportal area of adult cases (Fig. 3j). The interplay between these cell populations according to the severity of the fibrosis could be reflected in the profile shown by the IL-17A+/Foxp3+ cell ratio, although it is not statistically significant (Fig. 3k).

Finally, in the pediatric group concerning steatosis in the portal-periportal area, total lymphocyte count showed a profile tending to higher values with a more severe steatosis grade (Fig. 2o), while only CD4+ lymphocytes showed significantly higher values in cases with steatosis grade 3 (*p*=0.023) (Fig. 2p). In the intralobular area, CD8+ and CD4+ lymphocytes presented significantly higher values in cases with more severe steatosis (*p*=0.023 and *p*=0.012, respectively) (Fig. 2s). Most remarkable, only those severe cases displayed Foxp3+ and IL-17A+ cells (Fig. 2t); one possible explanation for this observation could be that CD4+ lymphocyte frequency is very low in steatosis grade 1 and 2 cases, therefore these subpopulations may be underrepresented in these stages. In adult cases, none of the lymphocyte populations analyzed showed a relationship with the steatosis degree (Fig. 3o–u). Interestingly, the pattern of CD4+ cells and their subpopulations mentioned for pediatric cases were not observed in the adult group (Fig. 3s,t). Nevertheless, it is interesting to mention that IL-17A+ cells were absent in cases with mild steatosis (Fig. 3t) with its consequent impact on the IL-17A+/Foxp3+ cell ratio (Fig. 3u).

An interesting final observation is that when NASH cases were considered separately, the same lymphocyte population profiles as those described for NAFLD, were observed (Supplementary Fig. 4 and 5); except for the CD8+ cell frequency in portal-periportal area, which was significantly increased in fibrosis ≥2 in adults (Supplementary Fig. 5). The latter could imply its possible participation in the fibrogenic process.

## Discussion

The immune system is one of the main drivers of NAFLD progression and other obesity-related comorbidities, where both the innate and adaptive immune systems are involved. T cells can be subdivided into multiple subsets with differential physiological functions, mainly CTL and various T helper subsets, but also several other subsets of T cells belonging to the innate rather than the adaptive immune system. Fatty liver may be more susceptible to injury because certain subsets of hepatic lymphocytes seem to be functionally and quantitatively altered^11^. Like in other hepatic diseases, Th17 cells play an important role not only mediating pathogen clearance but also contributing to tissue inflammatory responses and autoimmune disease progression in NAFLD. Interestingly, immune-mediated host injury can be also regulated by Treg cells which inhibit inflammation and maintain self-tolerance^11,17,22–24^. Tregs and Th17 cells share mechanisms and key mediators at the differentiation process, in this scenario, a combination of the immunoregulatory cytokine TGF-β and proinflammatory cytokine IL-6 inhibits Treg generation, elicits Th17 cell production and leads proinflammatory responses^17^. The reciprocal relationship between Treg and Th17 cells represents a delicate balance between tolerance and elicitation of immune responses^25^. Although many authors have studied the role of these populations in adult NAFLD, their association with liver damage is poorly discussed. Furthermore, little is known about liver immune response in pediatric population. This group represents a challenge itself because even in childhood, fatty liver can lead to cirrhosis and end-stage liver disease^26^. There are emerging data indicating that significant differences exist between adult and pediatric histological presentation; therefore, extrapolation of adult data may lead to a mistake^27^.

In this series of NAFLD it was shown that both Th and CTL were present, with different abundances, not only in the portal-periportal area but also in the intralobular zone. Th and CTL displayed similar frequencies in the portal-periportal area, while CTLs were the predominant cell population among the intralobular area. It is very interesting to compare this scenario of liver inflammation for a metabolic pathology with that observed for a chronic viral infection. In this regard, our group has also evaluated liver lymphocyte populations in HCV and HBV adult chronic infection demonstrating a characteristic profile for each pathology^22,23^. The median of the total lymphocyte count in NAFLD was at least three times higher than the one observed for HCV or HBV chronic infection denoting the great inflammatory activity, which is one of the three components, together with steatosis and lipotoxicity, of the vicious cycle of NAFLD pathogenesis (Supplementary Fig. 6).

Concerning Th subpopulation, there were some points to highlight, that is to say 1) the concomitant presence of Treg lymphocytes and CTL at the intralobular area may suggest an immunomodulatory interaction between them; 2) only few cases displayed intralobular Th17 cells, so it could be inferred that these cells could be exclusive of the portal-periportal area; and 3) Treg lymphocytes showed the highest frequency among the Th subpopulation in the portal-periportal area in adult cases but not in children. This result was strengthened by the case by case analysis of Treg and Th17. Worth mentioning the observed Treg predominance over Th17 in the portal-periportal area in adult cases also appeared in our previous studies on adult HCV or HBV chronic infection (Supplementary Fig. 6); however, in NAFLD patients it was more pronounced^22,23^. Therefore, the observed imbalance between Th17 and Treg cells may play an important role in the pathological mechanism and potential distinct progression of NAFLD. The Treg lymphocyte predominance together with the low Th17 lymphocyte frequency, delineates a skewed Th17/Treg balance towards Treg lymphocytes. However, none of these cell populations showed an association with the severity of liver damage *per se*. It should be borne in mind that since this study involved patient biopsies, a high variability in the results was found, which led to a detrimental impact on significant differences between the histological subgroups. However, Th17 and Treg cells demonstrated a delicate roleplay that could mirror their interrelationship and perhaps masked their actual role in the pathogenesis scenario. An example of this could be the inverted profile observed in adult patients in relation to inflammation (high inflammation, more Th17 frequency, less Treg, high Th17/Treg ratio).

Noteworthy, in the studied NAFLD cohorts the magnitude of the portal-periportal infiltrate seemed to be similar, in contrast to our previous findings in patients with chronic HCV infection, where pediatric cases exhibited a lower portal-periportal inflammatory infiltrate (total lymphocytes, CTL, and Th) than adult cases^28^. This discrepancy may be attributable to the differences in the disease severity distribution between pediatric and adult cases.

Interestingly, in pediatric cases a profile displaying lower counts of intralobular CTL accompanied by higher frequencies of Treg was observed in those cases with more severe inflammation. As mentioned above, this observation seems to reflect the immunomodulatory role of Treg over CTLs. Moreover, given the relationship between total portal-periportal lymphocytes and fibrosis severity, and despite the Th17 and Treg profiles mentioned above, we concluded that fibrogenesis could not exclusively be attributed to one of the cell population studied since all of them were at the liver infiltrate and may contribute to fibrosis activation and progression.

Another remarkable issue is that at the intralobular area, CTL and Th lymphocytes displayed significantly higher values in those cases with more severe steatosis and concomitantly only these cases exhibited Treg and Th17 cells. The Th frequency was very low in those cases with mild steatosis, so in consequence Treg and Th17 could not be identified, which may reflect the relationship between steatosis and the immune response. The presence of lymphocytes in the liver microenvironment may be a consequence of the lipid-induced hepatocyte stress, damage and cell death^29^.

It is worth mentioning that this study has some limitations. First, the liver biopsy has many drawbacks, such as sampling error, cost, and risk of complications and it is not of election in some NAFLD patients. Despite this, the biopsy is the gold standard for diagnosing progressive NASH and it is an invaluable specimen to easily determine and differentiate the location of infiltrating immune cells in the liver parenchyma in an accessible way for any pathology laboratory with no sophisticated equipment. In this sense liver infiltration assessment could complement routine histological diagnosis as a more precise approach with potential treatment implications. Second, although the number of samples included in our study seemed to be limited, it represents an important cohort, particularly the pediatric group and it has a number of cases similar to other studies^30–33^. It should be borne in mind that even today, there are no specific pediatric guidelines about the need and timing of a liver biopsy in pediatric NAFLD, in consequence the indication of a liver biopsy in children depends on the national consensus of local experts for each region. Concerning adults, another scenario is established, since the emergence of different less invasive markers of liver damage discourages the biopsy procedure, which in turn diminishes sample availability. Third, liver damage stages were not equally represented among children and adults, perhaps, due to the unethical concern of performing the biopsy procedure in a child with no apparently severe disease. This may be a critical point given that our observations may be influenced by differences in liver disease severity of each cohort. This drawback is difficult to overcome in a single center study due to the low availability of pediatric biopsies, perhaps a future larger multicenter project, with a larger number of cases representing all disease stages from each cohort, could validate and confirm our findings. Lastly, it should be pointed out that the liver microenvironment composition is dynamic along disease course, but our study design based on liver biopsy analysis, can only provide a snapshot of a time point. This issue could only be avoided with a longitudinal study including a paired liver biopsy approach that could explain the process in a more accurate way; however, it is nearly ethically unfeasible. Despite all the limitations pointed out above, the clinical relevance of a study like this remains in the observations and conclusions arose from the real liver disease scenario instead of an in vitro model. Moreover, the inclusion of pediatric liver biopsies to evaluate the role of intrahepatic infiltrating lymphocytes in NAFLD pathogenesis is a novel approximation of this study.

Besides, the importance of this research relay on the differences described in the infiltrate composition between histological areas; especially highlighting the characteristics of the portal-periportal infiltrate, an aspect that is not taken into account when performing the histological evaluation by applying NAFLD scoring system and that could have clinical significance if our observations are confirmed.

It must be stressed how immune response, especially CTL, Th17 and Treg, are involved in NAFLD pathogenesis. It is important to recognize the crucial role of Th17/Treg balance in the course of NAFLD not only in the pathogenesis of the disease, but also as a potential target for new therapies. In the era of precision medicine, patients with liver disease are expected to be diagnosed and treated according to their own "molecular signature" that ultimately allows the indication of "personalized medications". Hence, the evaluation and characterization of the inflammatory infiltrate may operate as a complement for the histological diagnosis which could even add precision to it. Consequently, it might be a future relevant research goal with a potential impact on clinical practice.

## Materials and methods

### Patients and samples

Cases with NAFLD presumptive diagnosis with liver echogenicity that correlated to hepatic steatosis or with metabolic risk factors (i.e. obesity or metabolic syndrome) were included. Excluding criteria were other causes of liver disease [autoimmune, genetic or endocrine diseases, hepatocellular carcinoma (HCC), HCV, HBV and/or HIV infection] and alcohol ingestion greater than 30g/day for men and 20 g/day for women.

Formalin-fixed and paraffin-embedded (FFPE) liver samples were collected retrospectively, based on the availability of enough material, from the archives at Pathology Division, Ricardo Gutierrez Children’s Hospital (from 2009 to 2018) and Italian Hospital of Buenos Aires (from 2010 to 2016).

This study has the approval of the Ethic Board of Ricardo Gutierrez Children Hospital and is in accordance with the 1964 Declaration of Helsinki and its later amendments. A written informed consent was obtained from each patient and from parents of pediatric patients prior to their inclusion in the study.

Clinical and histological features of patients are described in Table 1. Tables S2 and S3 show detailed clinical and histological features of each NAFLD patient.

## Histological analysis

To minimize inter-observer errors, one of the pathologists reviewed all the cases. Routine hematoxylin-eosin and Masson’s trichrome stain were applied on formalin-fixed paraffin-embedded liver biopsies to assess the histological diagnosis following the NAFLD scoring system^9^. A 9-point scale (steatosis = 0-3; lobular inflammation = 0-3; ballooning = 0-2) was used to weigh activity grade; in this sense, a score ≥5 corresponds to “definitive NASH”, 3-4 to “borderline NASH”, and ≤2 to “not NASH or simple steatosis”. Meanwhile, the evaluation of fibrosis was based on a 6-point scale defined as 1a, b = mild (1a)/ moderate (1b) zone 3 perisinusoidal fibrosis; 1c = only portal fibrosis; 2 = zone 3 and portal/ periportal fibrosis, 3 = bridging fibrosis, 4 = cirrhosis. Significant fibrosis was assumed when the fibrosis score was ≥ 2.

## Characterization of the inflammatory liver infiltrate

Based on the availability of enough biopsy material, infiltrate characterization was performed as previously describe^22,23^. The frequency and localization of the following lymphocyte populations and subpopulations were assessed: CTL (CD8+), Th (CD4+), Treg (Foxp3+) and Th17 (IL-17A+) using appropriate antibodies [rabbit anti-CD8 (Cat#790-4460, SP57, ready to use, VENTANA, Roche, Basel, Switzerland), rabbit anti-CD4 (Cat#790- 4423, SP35,ready to use, VENTANA, Roche, Basel, Switzerland), mouse anti-FoxP3 (Cat#ab20034, 236A/E7, 1:100, Abcam, Cambridge, UK) and goat anti-IL-17A (Cat#AF-317, AF-317-NA, 1:100, R&D Systems, Minneapolis, MN, USA)].

FFPE tissue slices were dewaxed and rehydrated by passing through an alcohol gradient by the conventional method. Permeabilization and epitope retrieval were performed using sodium citrate buffer (0.01 mol/L, pH 6) in autoclave (20 psi) for 5 minutes. Then, sections were incubated with primary antibody: 30 minutes at 37°C for CD8 and CD4, 1 hour at 25°C for anti-FoxP3 and 18 hours at 4°C for anti-IL-17A. The staining procedure was performed, according to the manufacturer's instructions, by applying VECTASTAIN ABC- HRP Kit (Cat#PK-4000, Vector Laboratories Inc, Burlingame, CA, USA) for both CD8 and CD4, the PolyTek HRP anti- Mouse Polymerized Imaging System (PIR080, ScyTek Laboratories, Logan, USA) for FoxP3, and the Cell & Tissue Staining Goat Kit (Cat#CTS008, HRP- DAB System, R&D Systems, Minneapolis, MN, USA) for IL-17A. PBS (Na2KPO4 10 mmol/L, KH2PO4 1.8 mmol/L, NaCl 137 mmol/L, KCl 2.6 mmol/L, pH 7.4) was used as washing buffer. Tonsil sections were used as positive controls as well as negative controls. For localization analysis portal-periportal area were defined as portal tract and acinar zone 1, and intralobular area corresponded to acinar zone 2 and 3. For quantitative analysis, both immunostained and total portal-periportal lymphocytes were counted in all portal tracts of each tissue section (400×), and then frequencies were calculated as positive/total lymphocytes of the whole specimen. Intralobular immunostained lymphocytes were also counted in 10 random fields (400×), and frequencies were informed as positive lymphocytes/10 fields. The number of total lymphocytes was obtained as the mean of total lymphocytes counted in each case for each labeled antigen.

## Statistical analysis

GraphPad Prism version 5.01 (GraphPad Software for Windows, San Diego, CA, USA, www.graphpad.com) was used. Outliers were examined by Grubb’s test. The comparison of means between groups normally distributed were done with ANOVA or Student’s t test; while Mann-Whitney U test or Kruskal Wallis test were applied to compare medians between groups not normally distributed.

The results shown in figures are depicted in scatter dot plot where the horizontal lines represent mean and SD.

Those cases with no Foxp3+ lymphocyte counts (Foxp3=0) were excluded in the IL-17A+/Foxp3+ cell ratio analysis in order to avoid indeterminacy.

## Supplementary Information

Supplementary Information
