# Supplementary material for: Hepatic lymphocytes involved in the pathogenesis of pediatric and adult non-alcoholic fatty liver disease
Source: Sci Rep. 2021 Mar 4;11:5129. doi: 10.1038/s41598-021-84674-z (PMC7933421; doi:10.1038/s41598-021-84674-z)
Supplement: Supplementary file 1 — Supplementary Information [file 41598_2021_84674_MOESM1_ESM.docx]

**Supplementary information**

**Hepatic lymphocytes involved in the pathogenesis of pediatric and adult non-alcoholic fatty liver disease**

Victoria Cairoli^1^, Elena De Matteo^1^, Daniela Rios^1^, Carol Lezama C^2^, Marcela Galoppo M^2^, Paola Casciato^3^, Eduardo Mullen^4^, Cecilia G Giadans^1^, Gustavo Bertot^5^, María Victoria Preciado^1^, Pamela Valva^1^.

^1^ Multidisciplinary Institute for Investigation in Pediatric Pathologies (IMIPP), CONICET-GCBA, Laboratory of Molecular Biology, Pathology Division, Ricardo Gutiérrez Children’s Hospital, CABA, Buenos Aires, C1425EFD, Argentina.

^2^ Liver Unit, Ricardo Gutiérrez Children’s Hospital; CABA, Buenos Aires, C1425EFD, Argentina.

^3^ Liver Unit, Italian’s Hospital of Buenos Aires; CABA, Buenos Aires, C1199 ABH, Argentina.

^4^ Pathology Division, Italian’s Hospital of Buenos Aires; CABA, Buenos Aires, C1199 ABH, Argentina.

^5^ H.A. Barceló Foundation-Medicine University, CABA, Buenos Aires, C1425EFD, Argentina.

**
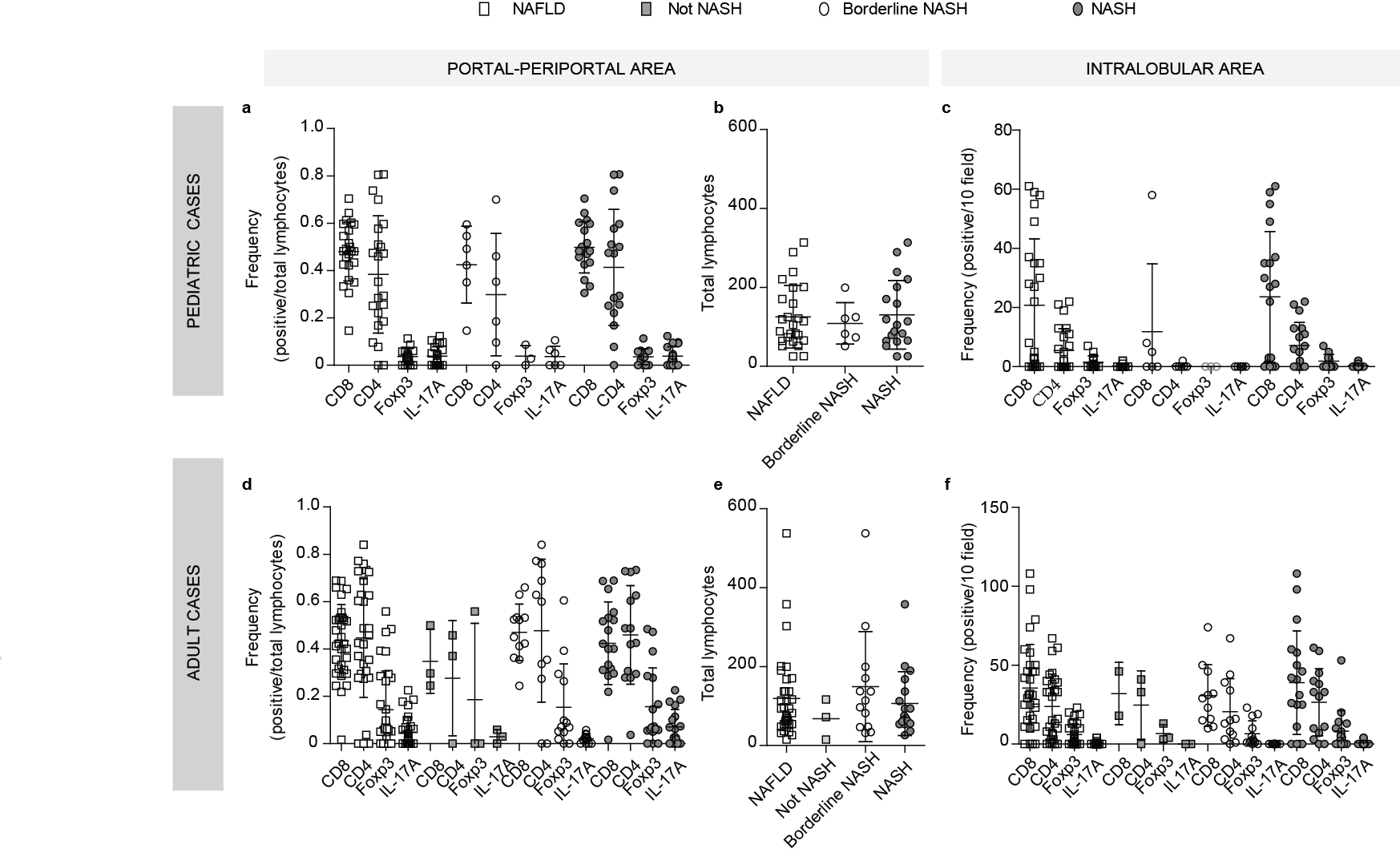

Supplementary figure 1: Frequencies of lymphocytes population.** Lymphocyte quantification from pediatric (a-c) and adult (d-f) cases. CD8+, CD4+, Foxp3+ and IL-17A+ cell frequency (a, d) and total lymphocytes (b, e) in portal-periportal area. CD8+, CD4+, Foxp3+ and IL-17A+ cell frequency of intralobular area (c, f). NAFLD refers to the entirety of the cases evaluated. NAFLD scoring system ≥5 corresponds to “NASH”, 3-4 to “borderline NASH”, and ≤2 to “not NASH or simple steatosis”.

**
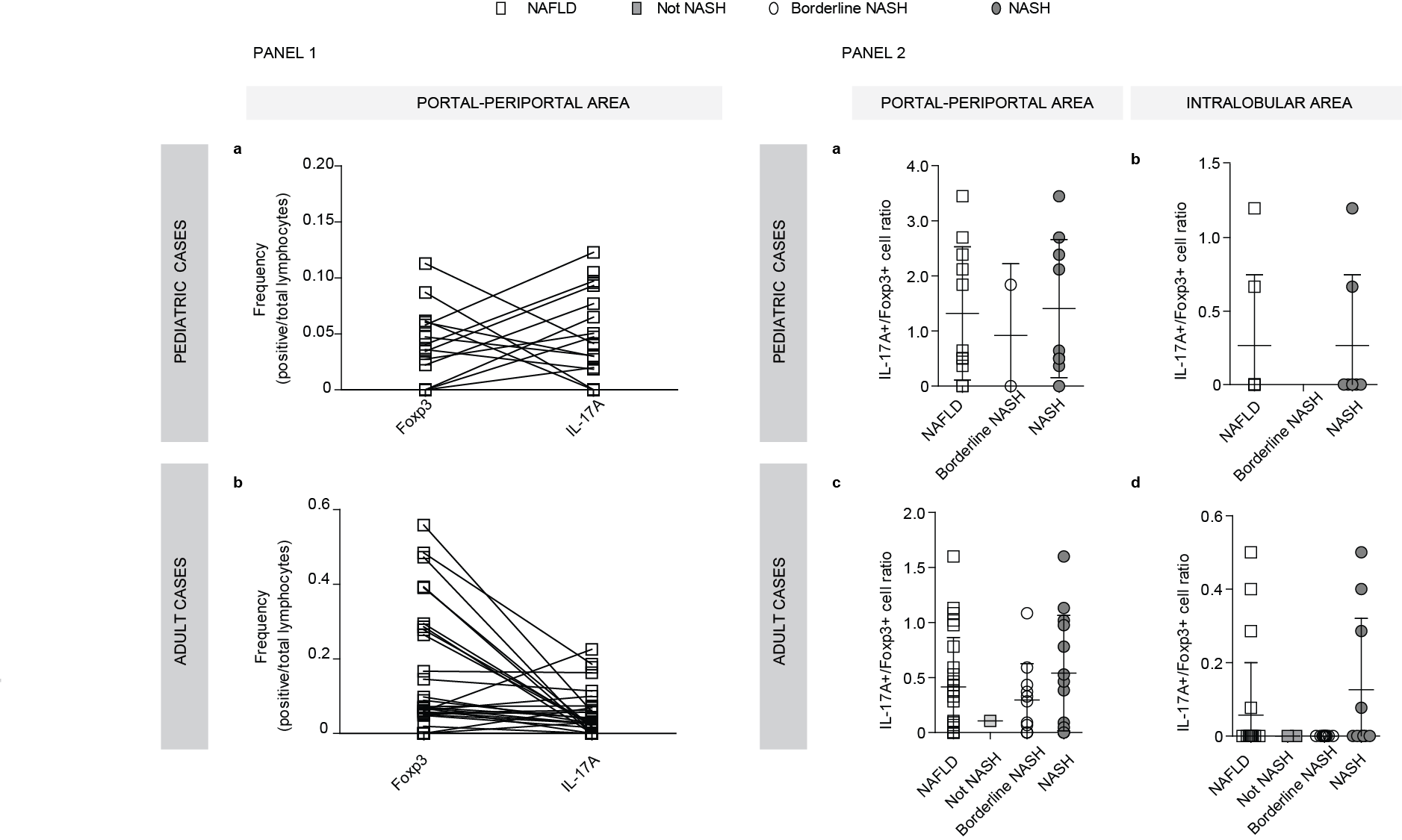
**

**Supplementary figure 2: PANEL 1. Frequencies of Foxp3+ and IL-17A+ cells from NAFLD patients.** Case by case Foxp3+ and IL-17A+ cell frequency in portal-periportal area from pediatric (a) and Adult (b) patients. **PANEL 2. IL-17A+/Foxp3+ cell ratio.** Results from pediatric (a-b) and adult (c-d) cases in portal-periportal (a, c) and intralobular area (b, d). NAFLD refers to the entirety of the cases evaluated. NAFLD scoring system ≥5 corresponds to “NASH”, 3-4 to “borderline NASH”, and ≤2 to “not NASH or simple steatosis”.

**
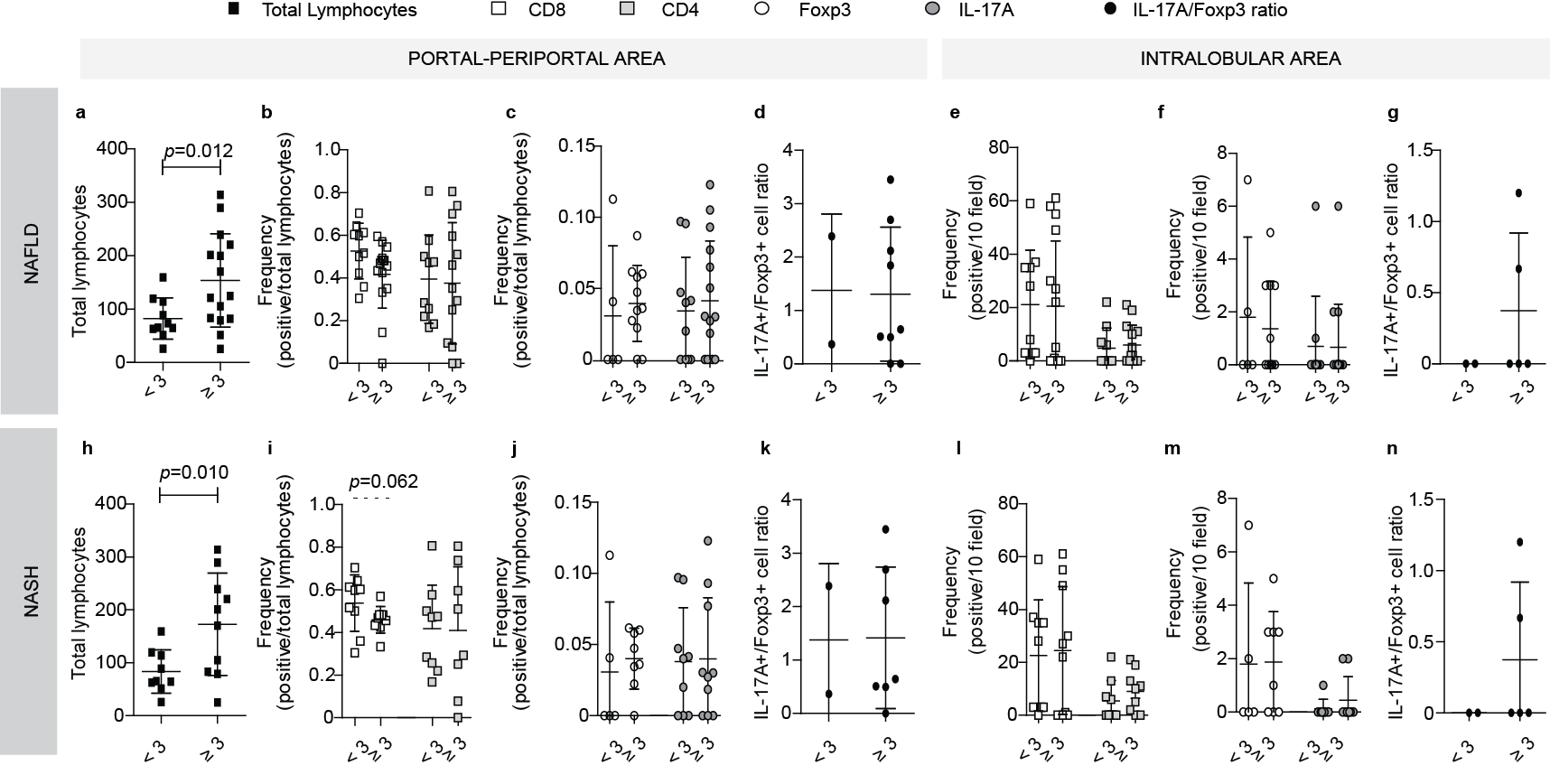

Supplementary figure 3: Relationship between intrahepatic infiltrate and advanced fibrosis in NAFLD pediatric cases.** Total lymphocytes (a), CD8+ and CD4+ (b), Foxp3+ and IL-17A+ cell frequency (c) and IL-17A+/Foxp3+ cell ratio (d) in portal-periportal area. CD8+ and CD4+ (e), Foxp3+ and IL-17A+ cell frequency (f) and IL-17A+/Foxp3+ cell ratio (g) in intralobular area. **
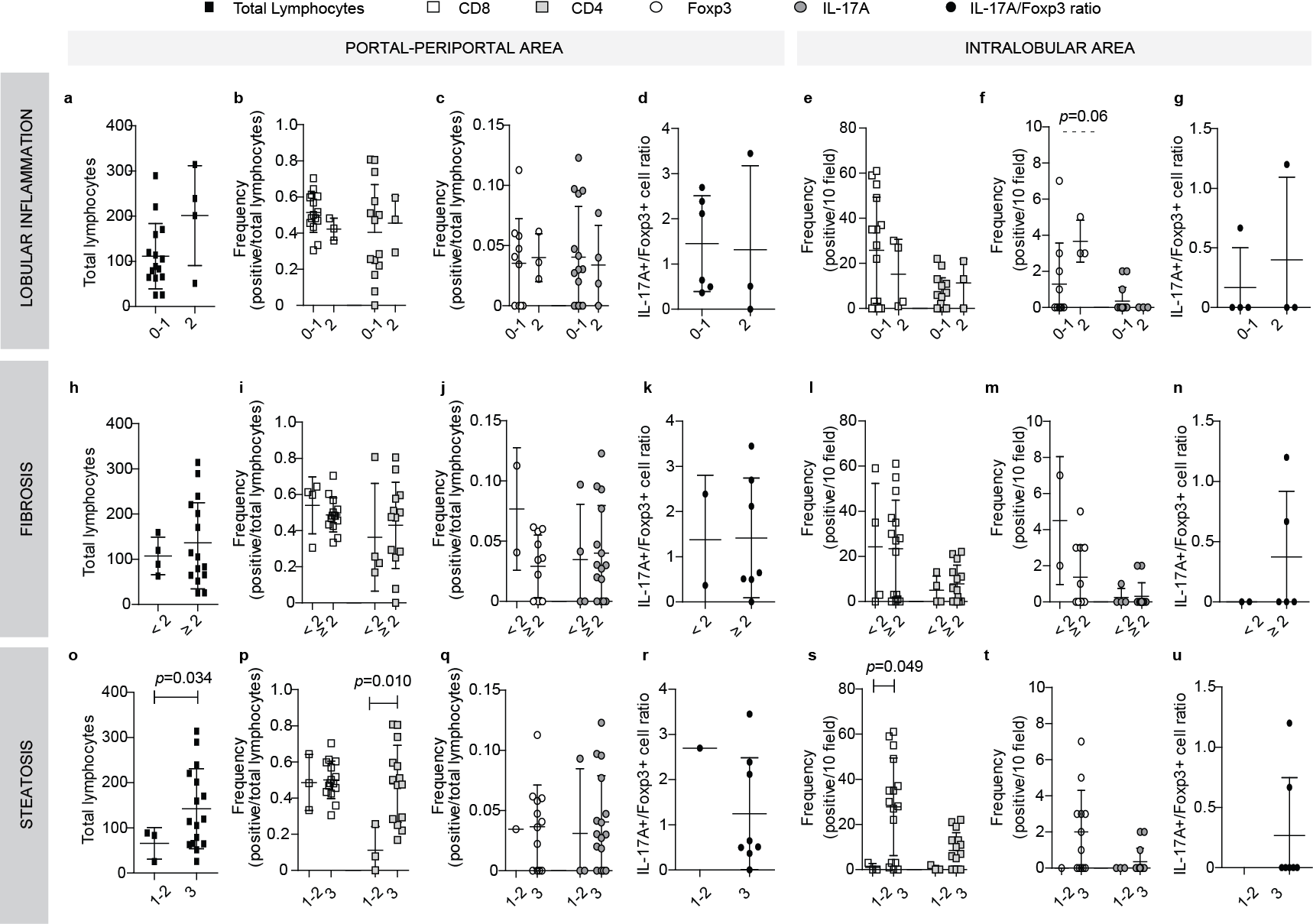

Supplementary Figure 4: Relationship between intrahepatic infiltrate and liver damage in NASH pediatric cases.** Lymphocyte quantification related to lobular inflammation (a-g), fibrosis (h-n) and steatosis (o-u) severity. Total lymphocytes (a, h, o), CD8+ and CD4+ (b, i, p), Foxp3+ and IL-17A+ cell frequency (c, j, q) and IL-17A+/Foxp3+ cells ratio (d, k, r) in portal-periportal area. CD8+ and CD4+ (e, l, s), Foxp3+ and IL-17A+ cell frequency (f, m, t) and IL-17A+/Foxp3+ cell ratio (g, n, u) in intralobular area.

**
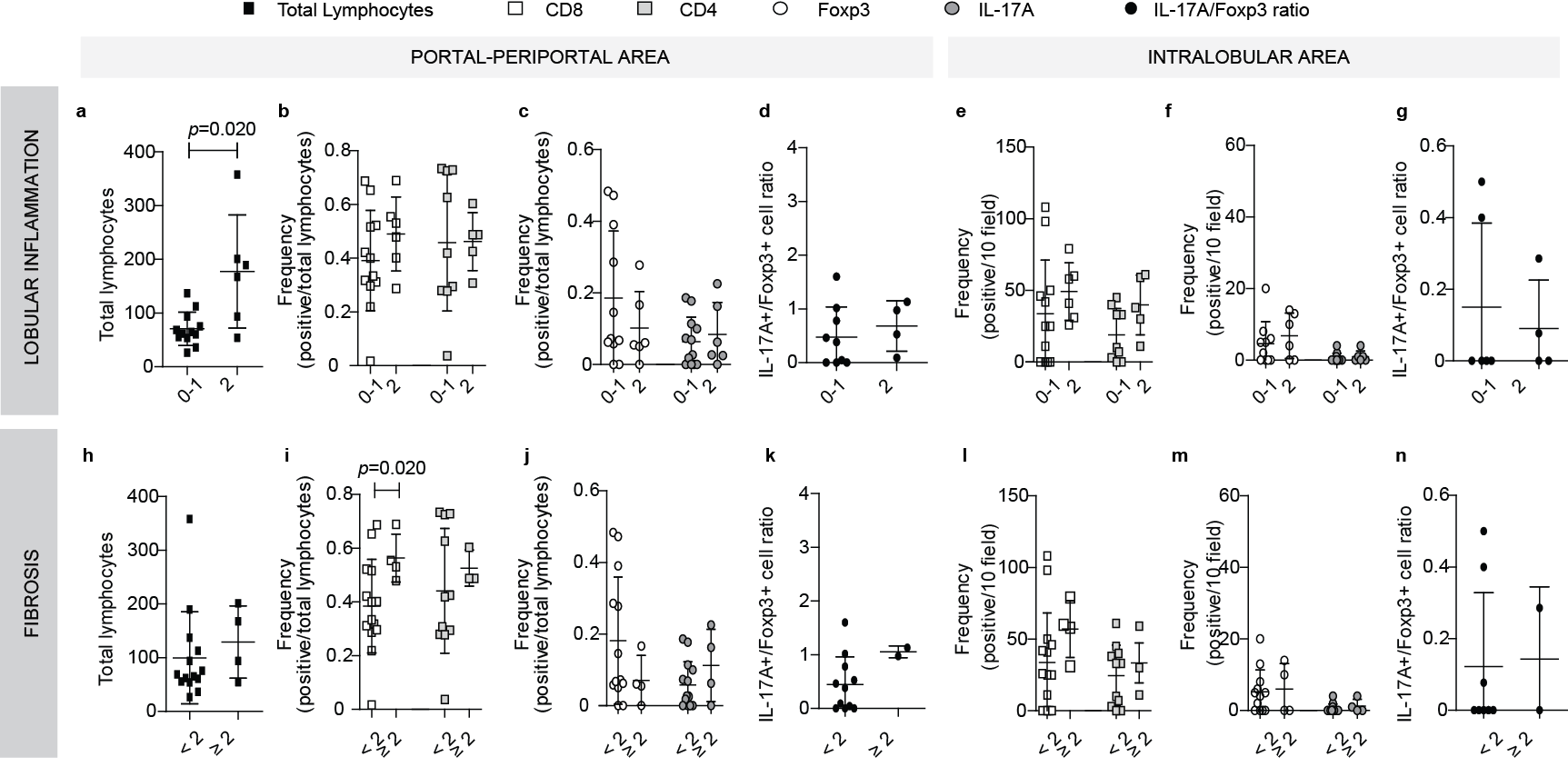
**

**Supplementary Figure 5: Relationship between intrahepatic infiltrate and liver damage in NASH adult cases.** Lymphocyte quantification related to lobular inflammation (a-g) and fibrosis (h-p) severity. Total lymphocytes (a, h), CD8+ and CD4+ (b, i), Foxp3+ and IL-17A+ cell frequency (c, j) and IL-17A+/Foxp3+ cell ratio (d, k) in portal-periportal area. CD8+ and CD4+ (e, l), Foxp3+ and IL-17A+ cell frequency (f, m) and IL-17A+/Foxp3+ cell ratio (g, n) in intralobular area.


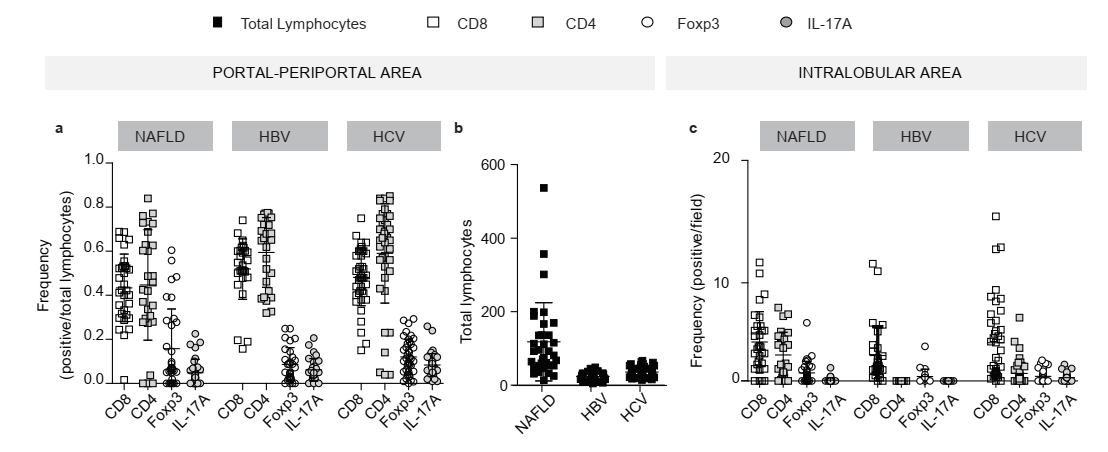


**Supplementary Figure 6: Frequencies of lymphocytes population from NAFLD, HBV and HCV cases.** CD8+, CD4+, Foxp3+ and IL-17A+ cell frequency (a) and total lymphocytes (b) in portal-periportal area. CD8+, CD4+, Foxp3+ and IL-17A+ cell frequency of intralobular area (c). Intralobular immunostained lymphocytes frequencies were informed as positive lymphocytes/field.

|  | **Pediatric patients** | **Adult patients** |
| --- | --- | --- |
| **Not NASH or simple steatosis** | |  |
| **Portal and periportal area** | |  |
| Total lymphocytes |  | 72 (15-116) |
| CD8^+^ cells |  | 0.296 (0.245-0.500) |
| CD4^+^cells |  | 0.370(0-0.457) |
| Foxp3^+^ cells |  | 0 (0-0.581) |
| IL-17A^+^ cells |  | 0.044 (0.029-0.058) |
| IL-17A^+^/Foxp3^+^ cell ratio |  | 0.105 (0-0.105) |
| **Intralobular area** |  |  |
| CD8^+^ cells |  | 32 (18-46) |
| CD4^+^cells |  | 33 (0-41) |
| Foxp3^+^ cells |  | 4 (3-13) |
| IL-17A^+^ cells |  | 0 |
| IL-17A^+^/Foxp3^+^ cell ratio |  | 0 |
| **Portal and periportal area** |  |  |
| Total lymphocytes | 102 (51-119) | 114 (32-538) |
| CD8^+^ cells | 0.457 (0.146-0.595) | 0.486 (0.244-0.659) |
| CD4^+^cells | 0.269 (0-0.700)  )=) | 0.600 (0-0.840) |
| Foxp3^+^ cells | 0.027 (0-0.087) | 0.070 (0-0.605) |
| IL-17A^+^ cells | 0.065 (0.50-0.105) | 0.027 (0.016-0.057) |
| IL-17A^+^/Foxp3^+^ cell ratio | 0 | 0.329 (0-1.084) |
| **Intralobular area** |  |  |
| CD8^+^ cells | 2.5 (0-58) | 29 (10-74) |
| CD4^+^cells | 0 (0-2) | 14(0-67) |
| Foxp3^+^ cells | 0 | 2 (0-23) |
| IL-17A^+^ cells | 0 | 0 |
| IL-17A^+^/Foxp3^+^ cell ratio | - | - |
| **Definitive NASH** |  |  |
| **Portal and periportal area** |  |  |
| Total lymphocytes | 105 (25-314) | 72 (26-358) |
| CD8^+^ cells | 0.482 (0.305-0.704) | 0.411 (0.016-0.689) |
| CD4^+^cells | 0.472 (0-0.806) | 0.452 (0.037-0.733) |
| Foxp3^+^ cells | 0.038 (0-0.112) | 0.069 (0-0.484) |
| IL-17A^+^ cells | 0.041 (0.018-0.123)  123)) | 0.0735 (0.018-0.225) |
| IL-17A^+^/Foxp3^+^ cell ratio | 0.644 (0-3.446) | 0.465 (0-1.600) |
| **Intralobular area** |  |  |
| CD8^+^ cells | 27 (0-61) | 36 (0-108) |
| CD4^+^cells | 5.5 (0-22) | 31.5 (0-61) |
| Foxp3^+^ cells | 1 (0-7) | 4.5 (0-20) |
| IL-17A^+^ cells | 0 (0-2) | 0 (0-4) |
| IL-17A^+^/Foxp3^+^ cell ratio ratio -17A/Foxp3 cellsratio | 0 (0-1.2) | 0 (0-0. 5) |

**Table S1: Lymphocyte frequencies in liver samples from “Not NASH or simple steatosis”, “Borderline NASH” and “Definitive NASH” cases.** Results are expressed as median (min.-max.). NAFLD scoring system ≥5 corresponds to “NASH”, 3-4 to “borderline NASH”, and ≤2 to “not NASH or simple steatosis”.

|  |  |  | | **Clinical and serological characteristics** | | | | | | | |  | | **Histological characteristics** | | | |
| --- | --- | --- | --- | --- | --- | --- | --- | --- | --- | --- | --- | --- | --- | --- | --- | --- | --- |
| **Patient** | **Gender** | **Age**  (ys) | **Lipid profile** | | | **Transaminases** | | | **BMI**  (kg/m^2^) |  | **Steatosis** | | **Lobular inflammation** | | **Ballooning** | **NAFLD activity score** | **Fibrosis** |
|  |  |  | Cholesterol (mg/dl) | | Triglycerides (mg/dl) | AST (UI/l) | ALT (UI/l) | AST/ALT ratio |  |  |  |  |  |  |  |  |  |
| 1 | M | 10 | 186 | | ND | 36 | 39 | 0.923 | 27.06 |  | 3 | | 2 | | 1 | 6 | 3 |
| 2 | M | 12 | 138 | | 91 | 83 | 157 | 0.529 | 26.53 |  | 3 | | 1 | | 1 | 5 | 2 |
| 3 | M | 8 | ND | | ND | 30 | 39 | 0.769 | 30.60 |  | 3 | | 2 | | 2 | 7 | 3 |
| 4 | M | 10 | 105 | | ND | 42 | 57 | 0.737 | 31.15 |  | 3 | | 1 | | 1 | 5 | 1 |
| 5 | M | 9 | 88 | | ND | 125 | 171 | 0.731 | 25.97 |  | 3 | | 3 | | 1 | 7 | 3 |
| 6 | M | 11 | 138 | | 88 | 101 | 213 | 0.474 | 30.31 |  | 3 | | 1 | | 1 | 5 | 1 |
| 7 | M | 11 | ND | | ND | 64 | 118 | 0.542 | 23.91 |  | 3 | | 1 | | 1 | 5 | 3 |
| 8 | M | 17 | 224 | | ND | 43 | 108 | 0.398 | 34.60 |  | 3 | | 1 | | 1 | 5 | 2 |
| 9 | M | 11 | 105 | | 59 | 28 | 50 | 0.560 | 23.71 |  | 3 | | 1 | | 1 | 5 | 3 |
| 10 | F | 13 | 181 | | 137 | 19 | 25 | 0.760 | 23.33 |  | 1 | | 1 | | 1 | 3 | 3 |
| 11 | F | 12 | 169 | | 212 | 23 | 22 | 1.045 | 30.63 |  | 3 | | 1 | | 1 | 5 | 3 |
| 12 | M | 15 | 175 | | ND | 126 | 145 | 0.869 | 40.22 |  | 3 | | 1 | | 1 | 5 | 4 |
| 13 | F | 11 | 155 | | 185 | 32 | 38 | 0.542 | 25.97 |  | 1 | | 1 | | 1 | 3 | 3 |
| 14 | M | 10 | 198 | | ND | 69 | 179 | 0.384 | 26.17 |  | 3 | | 1 | | 1 | 5 | 3 |
| 15 | M | 12 | 154 | | ND | 17 | 15 | 1.133 | ND |  | 1 | | 1 | | 1 | 3 | 3 |
| 16 | F | 10 | 128 | | ND | 20 | 24 | 0.833 | 27.30 |  | 2 | | 1 | | 2 | 5 | 3 |
| 17 | M | 14 | 128 | | 28 | 17 | 16 | 1.062 | 24.81 |  | 3 | | 1 | | 2 | 6 | 2 |
| 18 | F | 9 | ND | | ND | 30 | 42 | 0.714 | 29.93 |  | 1 | | 1 | | 2 | 4 | 3 |
| 19 | F | 4 | ND | | ND | ND | ND | ND | ND |  | 3 | | 1 | | 2 | 6 | 2 |
| 20 | F | 12 | 166 | | 120 | 21 | 18 | 1.167 | 30.70 |  | 2 | | 1 | | 2 | 5 | 3 |
| 21 | M | 10 | 160 | | ND | 72 | 147 | 0.490 | 25.46 |  | 3 | | 1 | | 1 | 5 | 3 |
| 22 | M | 13 | 142 | | 96 | 31 | 22 | 1.409 | 28.97 |  | 2 | | 0 | | 2 | 4 | 3 |
| 23 | M | 14 | ND | | 104 | 36 | 86 | 0.419 | 31.19 |  | 3 | | 1 | | 1 | 5 | 1c |
| 24 | F | 16 | ND | | ND | ND | ND | ND | ND |  | 3 | | 3 | | 1 | 7 | 2 |
| 25 | M | 15 | 211 | | 127 | 23 | 20 | 1.150 | 26.36 |  | 2 | | 1 | | 1 | 4 | 2 |
| 26 | F | 15 | 139 | | 37 | 16 | 24 | 0.667 | 34.77 |  | 2 | | 1 | | 2 | 5 | 1c |

**Table S2: Clinical and histological features of each NAFLD pediatric patient.**F: female, M: male. ND: no data. BMI: Body Mass Index, the values defined by Argentine Society of Pediatrics were used determine overweight and obesity (https://www.sap.org.ar/docs/profesionales/percentilos/completo.pdf). ALT: alanine aminotransferase; AST: aspartate aminotransferase. Normal ALT and AST levels for pediatric patients were ≤32 and ≤48 IU/L, respectively when testing was done at 37ºC. The normal ranges for cholesterol and triglyceride were 120-219 mg/dl and 31-119 mg/dl, respectively. Steatosis Grade: score 0 (<5%cells), 1 (5-33%), 2 (33-66%) and 3 (>66%); lobular inflammation: score 0 (0 foci), 1 (<2 foci), 2 (2-4 foci) and 3 (>4 foci); ballooning grade: score 0 (none), 1 (few ballooning cells) and 2 (many cells/prominent cells); fibrosis stage: score 1 (a, b = mild (1a)/ moderate (1b) zone 3 perisinusoidal fibrosis; 1c = only portal fibrosis); 2 (zone 3 and portal/ periportal fibrosis), 3 (bridging fibrosis) and 4 (cirrhosis).

|  |  |  | | **Clinical and serological characteristics** | | | | | | | |  | **Histological characteristics** | | | | |
| --- | --- | --- | --- | --- | --- | --- | --- | --- | --- | --- | --- | --- | --- | --- | --- | --- | --- |
| **Patient** | **Gender** | **Age**  (ys) | **Lipid profile** | | | **Transaminases** | | | **BMI**  (kg/m^2^) |  | **Steatosis** | | | **Lobular inflammation** | **Ballooning** | **NAFLD activity score** | **Fibrosis** |
|  |  |  | Cholesterol (mg/dl) | | Triglycerides (mg/dl) | AST (UI/l) | ALT (UI/l) | AST/ALT ratio |  |  |  |  |  |  |  |  |  |
| 1 | F | 45 | 186 | | 175 | 112 | 127 | 0.882 | 37.44 |  | 3 | | | 2 | 2 | 7 | 2 |
| 2 | F | 49 | 145 | | 60 | 26 | 54 | 0.481 | 33.30 |  | 2 | | | 1 | 1 | 4 | 0 |
| 3 | F | 72 | 200 | | 63 | 65 | 81 | 0.802 | 39.04 |  | 1 | | | 1 | 2 | 4 | 3 |
| 4 | M | 50 | 147 | | 103 | 35 | 75 | 0.467 | 35.08 |  | 2 | | | 1 | 2 | **5** | 1c |
| 5 | F | 58 | 165 | | 274 | 22 | 31 | 0.710 | 36.05 |  | 2 | | | 1 | 1 | 4 | 0 |
| 6 | F | 61 | 197 | | 84 | 80 | 90 | 0.889 | 27.47 |  | 3 | | | 0 | 0 | 3 | 1 |
| 7 | F | 46 | 199 | | 158 | 46 | 43 | 1.070 | 31.25 |  | 3 | | | 1 | 2 | 6 | 0 |
| 8 | F | 53 | 191 | | 180 | 36 | 50 | 0.720 | 27.73 |  | 3 | | | 2 | 2 | 7 | 0 |
| 9 | M | 28 | 246 | | 107 | 92 | 250 | 0.368 | 32.37 |  | 2 | | | 1 | 1 | 4 | 0 |
| 10 | M | 35 | 200 | | 109 | 81 | 94 | 0.862 | 32.41 |  | 3 | | | 1 | 1 | 5 | 0 |
| 11 | M | 30 | 207 | | 156 | 86 | 204 | 0.422 | 30.04 |  | 1 | | | 0 | 0 | 1 | 0 |
| 12 | F | 42 | 256 | | 375 | 87 | 98 | 0.888 | 47.33 |  | 3 | | | 2 | 1 | 6 | 1a |
| 13 | M | 38 | 285 | | 201 | 29 | 70 | 0.414 | 30.12 |  | 1 | | | 0 | 0 | 1 | 0 |
| 14 | M | 32 | 141 | | 76 | 44 | 67 | 0.657 | 40.52 |  | 3 | | | 1 | 1 | 5 | 0 |
| 15 | F | 70 | 200 | | 200 | 55 | 73 | 0.753 | 28.58 |  | 1 | | | 1 | 1 | 3 | 3 |
| 16 | M | 58 | 260 | | 195 | 61 | 82 | 0.744 | 37.46 |  | 3 | | | 2 | 1 | 6 | 2 |
| 17 | M | 45 | 126 | | 128 | 36 | 49 | 0.735 | 36.90 |  | 3 | | | 2 | 1 | 6 | 2 |
| 18 | M | 32 | 241 | | 134 | 60 | 144 | 0.417 | 35.54 |  | 3 | | | 1 | 1 | 5 | 0 |
| 19 | F | 63 | 220 | | 92 | 46 | 54 | 0.852 | 30.06 |  | 1 | | | 1 | 1 | 3 | 0 |
| 20 | M | 54 | 207 | | 157 | 50 | 89 | 0.562 | 26.79 |  | 2 | | | 0 | 1 | 3 | 0 |
| 21 | M | 47 | 254 | | 465 | 40 | 60 | 0.667 | 29.27 |  | 2 | | | 0 | 0 | 2 | 0 |
| 22 | F | 57 | 206 | | 268 | 35 | 58 | 0.603 | 28.84 |  | 2 | | | 1 | 1 | 4 | 1c |
| 23 | F | 48 | 224 | | 176 | 56 | 98 | 0.571 | 30.49 |  | 2 | | | 1 | 1 | 4 | 0 |
| 24 | M | 30 | 212 | | 151 | 39 | 99 | 0.394 | 30.07 |  | 3 | | | 1 | 1 | 5 | 0 |
| 25 | F | 53 | 327 | | 263 | 102 | 227 | 0.449 | 31.42 |  | 3 | | | 1 | 1 | 5 | 0 |
| 26 | M | 54 | 238 | | 241 | 184 | 254 | 0.724 | 39.86 |  | 2 | | | 1 | 1 | 4 | 1a |
| 27 | M | 49 | 207 | | 391 | 38 | 67 | 0.567 | 32.10 |  | 3 | | | 0 | 1 | 4 | 0 |
| 28 | M | 72 | ND | | ND | 279 | 242 | 0.745 | 32.14 |  | 3 | | | 2 | 2 | 7 | 2 |
| 29 | M | 63 | 179 | | 234 | 35 | 67 | 0.522 | 27.54 |  | 3 | | | 1 | 1 | 5 | 0 |
| 30 | F | 41 | 265 | | 198 | 45 | 76 | 0.592 | 26.18 |  | 3 | | | 1 | 2 | 6 | 0 |
| 31 | M | 50 | 156 | | 265 | 26 | 81 | 0.321 | 26.55 |  | 3 | | | 1 | 2 | 6 | 0 |
| 32 | M | 54 | 278 | | 241 | 56 | 79 | 0.709 | 30.64 |  | 1 | | | 1 | 1 | 3 | 0 |
| 33 | F | 43 | 224 | | 178 | 54 | 91 | 0.593 | 26.40 |  | 3 | | | 1 | 1 | 5 | 0 |
| 34 | F | 50 | 178 | | 189 | 34 | 61 | 0.557 | 30.38 |  | 3 | | | 1 | 1 | 5 | 0 |

**Table S3: Clinical and histological features of each NAFLD adult patient**. F: female, M: male. ND: no data. BMI: Body Mass Index [normal weight (<25.0 Kg/m^2^), overweight (25.0-29.9 Kg/m^2^) and obesity (≥30 Kg/m^2^)]; ALT: alanine aminotransferase; AST: aspartate aminotransferase. Normal ALT and AST levels for adult patients were ≤40 and ≤42 IU/L, respectively when testing was done at 37ºC. The normal ranges for cholesterol and triglyceride were 120-219 mg/dl and 31-119 mg/dl, respectively. Steatosis Grade: score 0 (<5%cells), 1 (5-33%), 2 (33-66%) and 3 (>66%); lobular inflammation: score 0 (0 foci), 1 (<2 foci), 2 (2-4 foci) and 3 (>4 foci); ballooning grade: score 0 (none), 1 (few ballooning cells) and 2 (many cells/prominent cells); fibrosis stage: score 1 (a, b = mild (1a)/ moderate (1b) zone 3 perisinusoidal fibrosis; 1c = only portal fibrosis); 2 (zone 3 and portal/ periportal fibrosis), 3 (bridging fibrosis) and 4 (cirrhosis).
